# War and oncology: cancer care in five Iraqi provinces impacted by the ISIL conflict

**DOI:** 10.3389/fonc.2023.1151242

**Published:** 2023-05-05

**Authors:** Mac Skelton, Ahmed Khalid Al-Mash'hadani, Zahi Abdul-Sater, Mohammed Saleem, Saad Alsaad, Marwa Kahtan, Ahmed Hazim Al-Samarai, Ahmed Moyed Al-Bakir, Layth Mula-Hussain

**Affiliations:** ^1^ Institute of Regional and International Studies, American University of Iraq-Sulaimani, Sulaymaniyah, Iraq; ^2^ Global Oncology Group, King's College London, London, United Kingdom; ^3^ Oncology Department, Anbar Cancer Center, Ramadi, Iraq; ^4^ Global Health Institute, American University of Beirut, Beirut, Lebanon; ^5^ College of Public Health, Phoenicia University, Mazraat El Daoudiyeh, Lebanon; ^6^ Medical Oncology Department, Kirkuk Oncology and Hematology Center, Kirkuk, Iraq; ^7^ Medical Oncology Department, Tikrit Oncology Center, Tikrit, Iraq; ^8^ Medical Oncology Department, Diyala Cancer Center, Baqubah, Iraq; ^9^ Medical Oncology Department, Specialized Oncology and Nuclear Medicine Hospital, Mosul, Iraq; ^10^ Radiation Oncology Department, Sultan Qaboos Comprehensive Cancer Care and Research Centre, Muscat, Oman; ^11^ Oncology Department, College of Medicine, Ninevah University, Mosul, Iraq

**Keywords:** cancer, oncology, conflict, Iraq war, Mosul, Islamic State, political economy, therapeutic geographies

## Abstract

War and cancer have been intertwined in Iraq for over three decades, a country where the legacies and ongoing impacts of conflict have been commonly associated with both increased cancer rates as well as the deterioration of cancer care. Most recently, the Islamic State of Iraq and the Levant (ISIL) violently occupied large portions of the country’s central and northern provinces between 2014 and 2017, causing devastating impacts on public cancer centers across central and northern Iraq. Focusing on the five Iraqi provinces previously under full or partial ISIL occupation, this article examines the immediate and long-term impacts of war on cancer care across three periods (before, during, and after the ISIL conflict). As there is little published data on oncology in these local contexts, the paper relies primarily upon the qualitative interviews and lived experience of oncologists serving in the five provinces studied. A political economy lens is applied to interpret the results, particularly the data related to progress in oncology reconstruction. It is argued that conflict generates immediate and long-term shifts in political and economic conditions that, in turn, shape the rebuilding of oncology infrastructure. The documentation of the destruction and reconstruction of local oncology systems is intended to benefit the next generation of cancer care practitioners in the Middle East and other conflict-affected regions areas in their efforts to adapt to conflict and rebuild from the legacies of war.

## 1 Introduction

War and cancer have long been intertwined in Iraq, a country where the legacies of conflict have been commonly associated with both increased cancer rates as well as the deterioration of cancer care (1–4). In addition to studies that explore the toxic remnants of war and potential impacts on cancer incidence (5–7), a growing body of research has explored the destructive impact of successive US-led wars on various aspects of cancer care, including cancer control (2), radiation oncology (3, 4), pediatric cancer care (8), and palliative cancer care (9). Protracted conflict has placed enormous burdens on cancer patients, many of whom have responded to the war-related deterioration of public oncology by travelling across provinces and international borders to pursue treatments in high-cost private centers (10, 11). These cross-border therapeutic geographies have generated catastrophic expenditures for families already suffering from displacement and the loss of livelihoods (12).

In this turn towards studying war and cancer care in the Iraqi context, what is notably lacking is an examination of how conflict generates different impacts on local cancer systems and hospitals depending on local particularities. The impact of war on oncology in Iraq has been examined as a general phenomenon at the national level. Though advances in oncology have been documented in specific areas of the country (13, 14), namely southern Iraq and the Kurdish region, no study has attempted to interrogate locally specific dynamics in the areas directly impacted by the Islamic State of Iraq and the Levant (ISIL) through a comparative political economy analysis. This lack of attention to local particularities has left policymakers and cancer practitioners with few tools to understand why oncology is relatively durable during conflict in some localities and not in others, and why oncology is rebuilt relatively quickly in some localities and not in others. Focusing on the five Iraqi provinces that were fully or partially overtaken by ISIL between 2014 and 2017, this article aims to shed light on how conflict generates locally distinct shifts in the distribution of cancer services, the availability of essential cancer pharmaceuticals and supplies, and the pace of oncology reconstruction. In addition, the article focuses on public oncology hospitals but avoids a strict public/private dichotomy. Indeed, war generates gaps in care filled by procedures or pharmaceuticals purchased in the emerging private sector or the black market.

**Map 1:**
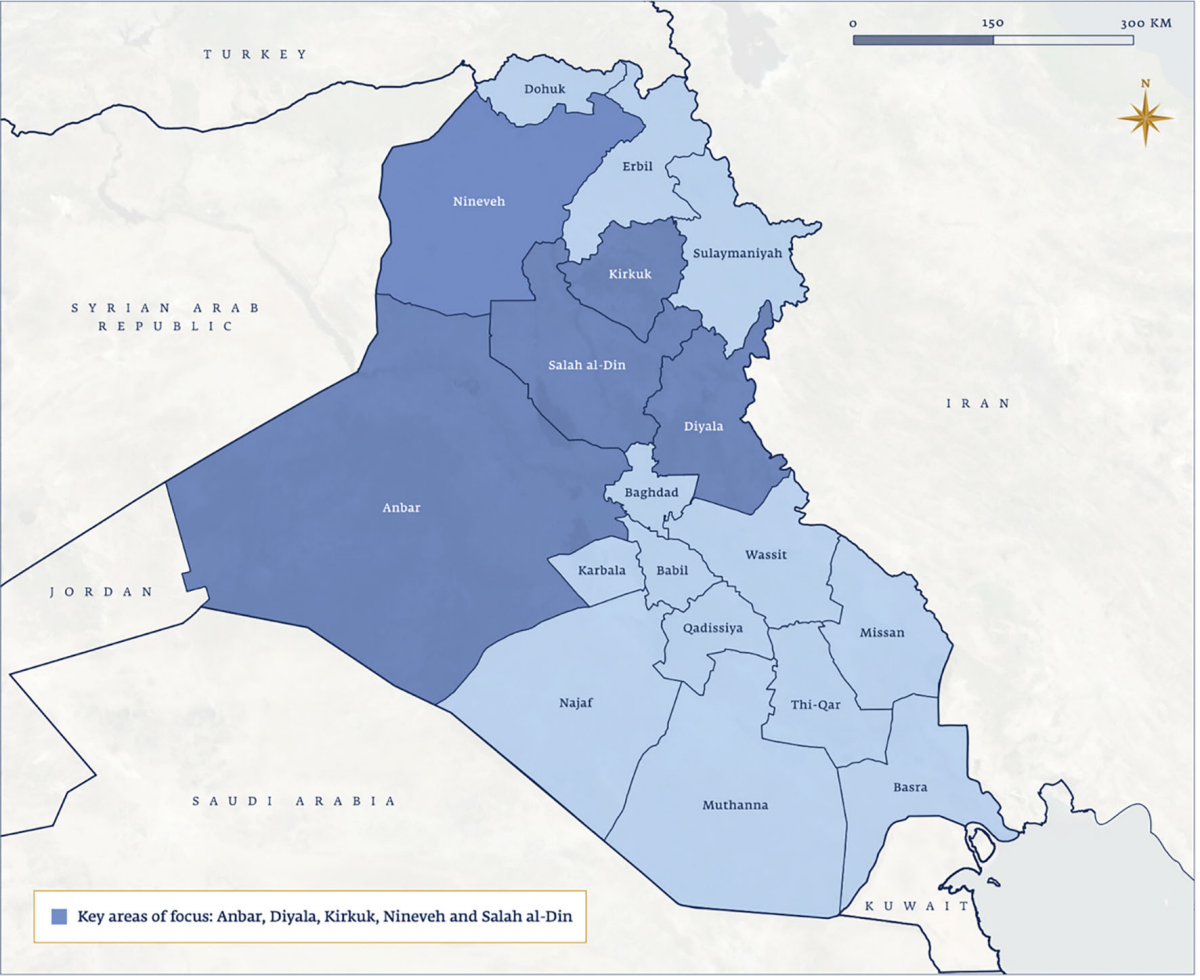
Provinces included in the study: Anbar, Diyala, Kirkuk, Nine-veh, Salah al-Din.

The primary focus of this article will be on the conflict period related to the rise and fall of the ISIL (2014-2017) and the reconstruction efforts in its aftermath (2017-2022). ISIL violently occupied large portions of the country’s central and northern provinces between 2014 and 2017, causing devastating impacts on a once robust healthcare system that was already severely damaged from the compounding effects of the first Gulf War, the UN embargo of the 1990s, and US-led invasion in 2003 and subsequent years of international military presence (15–17). The provinces most directly impacted throughout the ISIL period were Nineveh, Anbar, Kirkuk, Salah al-Din and Diyala (**Map 1**). The paper tells the story of oncology under conflict for each of the five provinces.

Relying on the accounts of oncologists who served during the ISIL period, the picture of oncology that emerges is geographically specific and highlights the unevenness of both conflict’s destructive impact and reconstruction’s benefits. For example, the ISIL conflict eviscerated oncology in some regions of Iraq while temporarily enhancing oncology resources in others; similarly, the reconstruction of cancer services has transpired rapidly in some provinces and languished in others. Understanding the factors that drive these varied outcomes in the distribution of care and the pace of reconstruction efforts can help shed light on the Iraqi situation and similar contexts where conflict is reshaping oncology, such as Ukraine (18). Importantly, the documentation of oncology under war is intended to benefit the next generation of cancer care practitioners in Iraq and other conflict-affected regions, who can learn from understanding how cancer services responded to previous periods of conflict.

## 2 Methods and limitations

This study examines the immediate and enduring impacts of conflict on oncology in the five provinces fully or partially occupied by ISIL between 2014 and 2017. Working chronologically across three periods (before, during, and after the ISIL conflict), the research elucidates the evolving impacts of war on the human resources, facilities, and available therapies necessary for cancer services. The chief aim of the analysis is to show the dynamic relationship between conflict and oncology in each of these provinces. In addition, a political economy lens is applied to interpret the results (19), particularly the data related to progress in oncology reconstruction. This is because conflict generates immediate and long-term shifts in political and economic conditions that, in turn, shape the rebuilding of medical infrastructure.

This article limits the scope of the analysis to the impact of war on the core infrastructural components of cancer care (e.g., the availability of pharmaceuticals, diagnostic tools, treatment facilities, and human resources). The crucial relational dimensions of cancer care (e.g., patient-doctor communication, levels of coordination and trust among all stakeholders, and patients’ perceptions of the quality of care) are not foregrounded in this analysis as these lines of inquiry would require an extensive, qualitative study centered around patients’ voices and experiences. The authors intend to publish such a patient-centered account in a forthcoming article.

This is a mixed methods paper primarily based on qualitative interviews and the lived experience of oncologists working in the affected provinces. An authorship team comprised of six Iraq-based oncologists from the five provinces was assembled (2 oncologists from Anbar; 1 from Nineveh; 1 from Salah al din; 1 from Diyala; and 1 from Kirkuk) in addition to three international colleagues with either research or practitioner experience in Iraq and the wider MENA region. During 3 group meetings in 2022, the authorship team shared and contrasted accounts of the three periods examined. A series of follow-up semi-structured interviews were conducted with each member of the Iraq-based authorship team. The combined authorship team analyzed the interview data collectively to generate further insights and points of comparison.

This data from the perspective of oncologists was supplemented by an earlier dataset collected in 2016 and 2017 during the height of the ISIL conflict, consisting of qualitative interviews with 40 cancer patients undergoing treatment in the public oncology hospitals of Erbil, Sulaymaniyah or Kirkuk who had fled one of the five provinces examined due to the rise of ISIL (10 from Nineveh; 10 patients from Anbar; 5 from the ISIL-occupied areas of southern Kirkuk; 10 from Salah al-Din; and 5 from Diyala). Though the core findings of the study are derived from the perspective of oncologists due to the focus on tracking shifts in cancer care infrastructure, the limited inclusion of the patient data in the analysis was necessary to verify or complicate the accounts of the co-authors/oncologists, particularly when assertions were made regarding the implications of shifting oncology capacity for patients’ access to cancer services.

## 3 Results

In the following sections, the interview data has been used to develop key trends in the evolving impact of conflict on the essential infrastructures of cancer care (e.g., facilities, pharmaceuticals, equipment, human resources) at the provincial level, focusing on (a) the status of oncology before the ISIL period; (b) the transformations of oncology during the period of severe conflict conditions (2014-2017); (c) the efforts and ongoing challenges to rebuilding cancer care between ‘liberation’ in 2017 and the present up to the time of writing in 2023. Three provinces (Nineveh, Anbar and Kirkuk) are explored in-depth. These three provinces receive extensive attention due to the significantly larger oncology infrastructure. On the other hand, Salah al-Din and Diyala are examined in an abbreviated fashion due to the relatively recent and limited development of oncology in those areas. **Table 1** summarizes the findings from the 5 provinces.

**Table 1:** Summary of findings: Cancer care in five provinces affected by the ISIL conflict.

	Before ISIL (Before 2014)	During ISIL (2014-2017)	After ISIL (2017-present)
**Nineveh**	Public oncology services began in 1979 and remained relatively comprehensive (including radiotherapy) over the course of multiple wars. Prior to 2014, there were 3 oncology centers and 8 specialists.	Nineveh witnessed a gradual deterioration of oncology services during the first 2 years of ISIL rule due to the disruption of supply lines; later oncology facilities were bombed/destroyed during liberation battles.	Specialists returned (11 total), but the province has witnessed a slow reconstruction of public oncology facilities. Services are currently limited to chemotherapy and basic diagnostics, with no functional radiotherapy.
**Anbar**	A single oncology unit was established in Ramadi in 2008 but saw slow progress (only 1 specialist), with a narrow range of cytotoxic and hormonal therapies.	The province witnessed a total shutdown of the Ramadi oncology unit after the ISIL takeover of the city, as all oncology nurses and staff were forced to flee.	Anbar witnessed a remarkably rapid reconstruction and expansion of oncology services (9 specialists, 2 centers). Anbar is becoming an oncology hub, but radiotherapy remains unavailable.
**Kirkuk**	A single oncology center was established in 2007 and services were steadily expanding (5 specialists by 2013). Radiotherapy was unavailable.	As ISIL failed to take over the capital of the province, Kirkuk’s oncology center became a haven for the displaced. Government funding increased to cover displaced cancer patients; thus, the availability of cancer drugs expanded during the conflict.	With supplementary war-time government support removed, Kirkuk witnessed a regression to pre-2014 levels of pharmaceuticals. Radiotherapy remains unavailable.
**Salah al-Din**	Limited oncology services were initiated in the provincial capital of Tikrit in 2008 under the direction of one oncologist. Radiotherapy was unavailable.	Tikrit’s oncology center was shut down as ISIL took over the city. Makeshift oncology services were delivered in Samarra city at minimum capacity throughout the ISIL occupation of Tikrit.	Cancer services have returned to Tikrit at limited levels, but the local government recently announced plans for a new oncology center. Radiotherapy remains unavailable till end of 2022.
**Diyala**	The province lacked oncology services. Patients sought cancer treatment in Baghdad, Sulaymaniyah, or Kirkuk.	No oncology services.	In the aftermath of liberation, a small oncology department was created in 2018 (1 oncologist). Chemotherapy approval was obtained in 2019, and a cancer center is currently under construction. Still, no radiotherapy.

### 3.1 Nineveh province (Mosul)

#### 3.1.1 Overview of trends: cancer and conflict

##### 3.1.1.1 Advanced oncology capacity before ISIL

During the 1980s, Nineveh gradually developed comprehensive public oncology services, including radiotherapy, and became the second largest hub for oncology in the country behind Baghdad. Cancer patients travelled to the provincial capital, Mosul city, from across northern/central Iraq. Despite the devastating impacts of the UN Sanctions (1991-2003) and US-led invasion and occupation on oncology (2003-2011), public oncology services remained comprehensive, boasting one of the few working radiotherapy machines in the country. A single new linear accelerator (linac) eventually replaced the two outdated cobalt radiotherapy machines.

##### 3.1.1.2 Gradual deterioration of oncology capacity during ISIL

ISIL took over Mosul city in June 2014. Though medical supply lines from Baghdad were soon cut, oncologists relied upon existing pharmaceutical stocks and equipment to maintain adequate cancer services for approximately one year. By the summer of 2015, the oncology apparatus began suffering from mass shortages in chemotherapy agents and the inability to maintain radiotherapy equipment. As a result, cancer services became very limited. In 2016-2017, liberation battles resulted in the cessation of oncology services; oncology facilities were ultimately bombed and destroyed.

##### 3.1.1.3 Slow reconstruction of oncology capacity after ISIL

Oncology services have commenced in makeshift facilities, but reconstruction of the public oncology facilities has proceeded at a glacial pace. Services are limited to chemotherapy and basic diagnostics, with no functional radiotherapy. An emerging private sector has started to fill the gaps in public services, creating additional costs for a population suffering from the war-related loss of livelihoods.

**Table:** 

	Before ISIL	During ISIL	After ISIL
Oncologists	8	3	11
Oncology facilities (public)	3	1	Makeshift temporary facilities
Radiotherapy machine	1	0	0

#### 3.1.2 Cancer care before ISIL

The Iraq-based oncologists contributing to this study all concurred that Mosul once represented the pinnacle of cancer care in Iraq. Since 1979, Mosul city has been home to the Specialized Oncology and Nuclear Medicine Hospital (SONMH). During the 1980s, Mosul developed comprehensive cancer services and became an oncology referral hub for Nineveh and nearby provinces (e.g., Erbil, Dohuk, Salah al-Din, Kirkuk). However, during the UN sanctions of the 1990s, severe restrictions on the import of chemotherapy agents and cobalt source replacement dramatically reduced the continuity and quality of care. In addition, the US-led invasion of 2003 ushered in a period of heightened political instability and insecurity in the province, compelling many medical professionals from Mosul to flee the country. As the intensity of the fighting reached a high point in 2006 and 2007, the relative stability of the nearby semi-autonomous Kurdistan Region – and its emerging oncology system in Erbil, Dohuk and Sulaymaniyah – led to a slight reduction in cross-provincial referrals to Mosul city.

Despite the challenges related to conflict and political instability, oncologists in Mosul city endeavored to maintain advanced public cancer services. In 2013, the year before the takeover of Mosul city by ISIL, public oncology services in Mosul included three centers (SONMH, Hematology Center, and Pediatric Oncology Center), which jointly had a capacity for over 60 beds, a pain and palliative care clinic, a colostomy care clinic, a radiotherapy department with a fully functioning linac, and a thyroid gland outpatient clinic. In sum, oncology services in pre-ISIL Nineveh were at a level of sophistication that far exceeded any other provinces examined in this paper. Mosul city was a national hub for oncology services.

#### 3.1.3 Cancer care during ISIL

The deterioration of oncology transpired gradually throughout the ISIL occupation of Mosul city. In June 2014, ISIL swiftly took over the entire city of Mosul, including the area where oncology services are located. Mosul’s highly experienced oncologists, who had endured repeated war and violence, treated this situation like any other. For the first several months of the ISIL occupation, cancer drugs generally remained available from the previous year’s stock of pharmaceuticals (e.g., Herceptin, Avastin, Erlotinib, Sunitinib, Pazopanib, etc.). ISIL did not actively obstruct basic cancer care delivery during the initial months. But problems were looming on the horizon.

Cut off from replenishing supplies from the Baghdad-based Ministry of Health (MoH), oncology hospitals were already witnessing severe shortages in chemotherapy and equipment by the spring and summer of 2015. The linac machine ultimately ceased functioning; the gamma camera stopped working; analgesics required for the palliative care clinic were no longer available. The public stock of chemotherapy agents was increasingly scarce and was almost empty by the beginning of 2016. Smuggling routes allowed chemotherapy agents to enter local circulation in Mosul’s pharmacies and the black market (at a cost that exceeded the ability of most patients). The cooling systems required to transport biological therapies were unavailable. By 2016, only three of the eight oncology specialists remained in the city, and the care they could provide was limited to the administration of chemotherapy purchased on the private market. Many patients attempted to flee the province and gain access to oncology centers elsewhere, but the exit routes were expensive and dangerous.

The commencement of the liberation battle in October 2016 injected new challenges in the delivery of oncology. The battle proceeded in two stages. First, the Iraqi Army and Coalition forces liberated the eastern side of the Tigris River as ISIL fighters retreated to the western side – the side of the city’s leading hospitals, including the SONMH. Mosul residents on the liberated eastern side were now effectively cut off from local cancer care, and many attempted to travel to Erbil with the help of WHO ambulances. Meanwhile, cancer services on the western side quickly became untenable. ISIL saw the medical apparatus as a strategic asset from the standpoint of providing care to wounded fighters, but oncology was regarded as irrelevant to sustaining a fighting force.

As the battle intensified, ISIL fighters expelled all doctors and staff from the SONMH, repurposing the facility as a military fortress. Oncologists and oncology nurses were then forced by ISIL to work in the surgical hospital to provide general medical services and serve as human shields against Coalition airstrikes. Ultimately the battle over the western side of Mosul city resulted in the aerial bombing of much of the medical infrastructure, including the hematology center and the SONMH. The once famous oncology apparatus, in operation continuously since 1979 through multiple periods of war and sanctions, was reduced to rubble in early 2017.

#### 3.1.4 Cancer care after ISIL

After the battle’s conclusion, the destruction on the western side of the city made any notion of reconstituting oncology services a distant prospect. Most of western Mosul’s buildings, houses, roads, electricity lines, and water treatment channels had been destroyed by aerial bombing and mortal shelling. The eastern side was relatively untouched by the effects of aerial bombing, however. Therefore, the decision was made to install a makeshift oncology unit on the eastern side. For several months, a small group of oncologists had two small rooms underneath a staircase and one bed for the administration of chemotherapy. In early 2018 they were able to expand this temporary facility into 10-15 beds and a patient lobby, and in 2020 they were able to expand further to 50 beds. But these expansions were made possible by installing temporary cabins repurposed from internally displaced people (IDP) camps, which are poorly ventilated and developed leaks that could not withstand the winter rains. Recently they moved out of the caravans into yet another temporary facility, which remains far too small to meet capacity. As a result, patients undergoing chemotherapy are stacked together tightly. None of the advanced technologies (e.g., gamma camera, linac machine) have been restored, and neither have the specialized clinics (e.g., pain clinic, colostomy clinic, palliative care clinic) been reconstituted.

To the time of this writing in December 2022, less than 20% of the SONMH has been completed. The impact of these severe limitations is that Mosul’s historical position as a center for oncology in Iraq has become increasingly diminished. As advanced diagnostics are only available in Dohuk, Erbil, and Baghdad, and radiotherapy in the latter two, these cities are now supplanting Mosul in the referral chain. Whereas Mosul attracted patients from across northern and central Iraq until 2014, today, most patients are local. Human resources are one bright spot. 11 oncologists are now serving in Mosul, compared to 8 in the pre-ISIL period. While this increase could furnish the ground for a bright future of oncology in Mosul, the status quo is unsustainable from the standpoint of infrastructure. Future generations of medical graduates will look elsewhere as long as oncology in Mosul is associated with makeshift delivery conditions. The discussion section will explore the political economic factors that have contributed to the slow pace of oncology reconstruction.

### 3.2 Anbar Province

#### 3.2.1 Overview of trends: cancer and conflict

##### 3.2.1.1 Minimal oncology capacity before ISIL

In 2008, the first oncology unit was opened in Ramadi Teaching Hospital. Cancer services remained limited during the subsequent years due to ongoing conflict and political instability in the province.

##### 3.2.1.2 Curtailment of oncology capacity during ISIL

In January 2014, ISIL took over Fallujah, the first Iraqi city to fall. In May 2014, ISIL took over half of Ramadi city, but the Iraqi military prevented ISIL from entering the area where the oncology unit is located. Limited cancer services continued. In April and May 2015, ISIL completed its takeover of Ramadi city, resulting in the shutting down of the oncology unit as specialists and nurses were forced to flee the city.

##### 3.2.1.3 Rapid expansion in oncology capacity after ISIL

Ramadi was liberated by the Iraqi military in February 2016. By May 2016, the cancer unit reopened in Ramadi Teaching Hospital with one specialist. Capacity quickly expanded. In 2018, an expanded oncology unit was opened in Ramadi Teaching Hospital (25 beds, 15 nurse staff, and 5 oncologists). An oncology unit in Fallujah was opened in 2019. In 2020, a fully staffed oncology center was opened in Ramadi (9 oncologists, 3 radiologists, 50 beds, and nearly 60 nurses).

**Table:** 

	Before ISIL	During ISIL	After ISIL
Oncologists	1	0-1	9
Oncology facilities (public)	1	0-1	2
Radiotherapy	0	0	0

#### 3.2.2 Cancer care before ISIL

Oncology is a recent development in Anbar province. According to the interviews with the Iraq-based oncologists conducted for this study, historically residents of Anbar sought cancer treatments outside the province in the public oncology centers of Baghdad due to the capital’s proximity. Despite concerns among citizens about increased cancer incidence potentially stemming from war-related environmental toxins, efforts to initiate oncology services in the province did not begin until 2008 with the establishment a public cancer unit within Ramadi Teaching Hospital. The unit had a capacity for 30 beds and was staffed by one oncologist and 9 nurses. Basic diagnostics and a limited range of cytotoxic and hormonal therapies were administered. A radiotherapy unit was under construction but never completed, meaning that all patients needing radiotherapy had to travel to Baghdad, Mosul, the Kurdistan Region, or abroad. Though cancer services remained minimal before ISIL, one crucial development was that seven oncology residents designated for service in Anbar began their training in 2010 abroad or other provinces. This fortuitous infusion of young trainees before the onset of the ISIL war would bode well for Anbar’s eventual reconstruction.

#### 3.2.3 Cancer care during ISIL

Oncologists from Anbar recounted how the province’s nascent cancer services were weakened and eventually collapsed during the ISIL war. Due to its multiple borders and permeability to foreign fighters, Anbar was the first Iraqi province to witness major fighting from ISIL and other militant groups. The provincial capital, Ramadi city – where the sole oncology unit was located – was partially overtaken in May 2014. Fortunately, the zone of the city hosting the oncology unit remained under the control of the Iraqi Army for almost 12 more months. Oncology services continued to be operational during that period. But in April 2015, ISIL commenced an onslaught to overtake the remainder of Ramadi. Nurses and the sole oncology specialist fled the city.

By May 2015, the ISIL takeover of Ramadi was complete, and oncology was no longer available in the city. While some previously diagnosed cancer patients could flee to other provinces to continue care, those who were forced to remain in Anbar during ISIL control went without treatment of any kind. Accessing chemotherapy agents and other drugs on the black market or private pharmacies was nearly impossible. Because the Iraqi military regarded the presence of ISIL in Anbar as a major security threat due to its proximity to Baghdad, security forces blockaded virtually all traffic of goods coming in and out of the province. Previously diagnosed cancer patients who survived those dark days in Anbar recall juggling a lack of medications and food and, consequently, the unchecked painful advance of their disease. Most did not survive. The seven oncology residents mentioned previously – all of whom were now displaced to other provinces or countries – did what they could to follow up with displaced patients from Anbar, helping them manage symptoms and clear bureaucratic hurdles in accessing care outside the province. Care outside of Anbar in provinces such as Baghdad, Kirkuk and Erbil were available in public oncology hospitals but nearly always involved significant wait times and expenditures due to shortages and gaps in service, placing a significant financial and practical burden on displaced patients.

#### 3.2.4 Cancer care after ISIL

After the liberation of Ramadi in February 2016, a limited set of oncology services returned to the city with the re-opening of the cancer unit in May 2016. The radiotherapy facility under construction had been destroyed in the fighting. The building housing cancer services had been badly damaged in the bombings and airstrikes, and space was minimal. The oncology unit dealt with dozens of advanced-stage patients who had not undergone adequate treatment during ISIL control – arriving with stacks of reports from multiple hospitals and often very unclear protocols. But conditions would rapidly improve due to favorable post-conflict political and economic conditions (see discussion section). In 2018, the Anbar Directorate of Health announced the opening an expanded cancer unit with 25 beds, 5 oncologists and 15 nursing staff. Then, in 2020, a stand-alone specialized oncology center was established, which replaced the previous cancer unit. The center boasted 9 oncologists, 3 radiologists, 2 laboratory specialists, and 60 nurses. The center has expanded diagnostic equipment, well-stocked chemotherapy agents, and laboratory apparatuses. Private oncology services are also rising in Anbar, including clinics offering chemotherapy, surgical oncology, and diagnostic and interventional radiology. The ongoing lack of radiotherapy continues to force Anbar residents to travel to Baghdad or abroad for that aspect of their treatment, however.

### 3.3 Kirkuk Province

#### 3.3.1 Overview of trends: cancer and conflict

##### 3.3.1.1 Oncology capacity before ISIL

In 2007, a public cancer department was established within Azadi General Hospital for diagnostics and basic chemotherapy (3 oncologists, 12 beds). Capacity was expanded between 2011 and 2013 (5 oncologists, 60 beds); more chemotherapy agents became available.

##### 3.3.1.2 Partial expansion of oncology capacity during ISIL

As ISIL never took over Kirkuk city, the number of cancer patients expanded as Kirkuk city became home to displaced cancer patients from the ISIL-occupied areas of southern Kirkuk and other provinces. Government funding increased for drugs/equipment to account for the higher patient load, but the cancer unit’s human resources did not change. The patient load decreased towards the end of the ISIL conflict due to the forced expulsion of Sunni Arab displaced persons from the city.

##### 3.3.1.3 Stagnation in oncology capacity after ISIL

After the liberation of southern Kirkuk in 2017, Kirkuk’s oncology capacity returned to pre-ISIL levels due to the removal of the additional government support provided during the conflict era.

**Table:** 

	Before ISIL	During ISIL	After ISIL
Oncologists	5	5	8
Oncology facilities (public)	1	1	1
Radiotherapy	0	0	0

#### 3.3.2 Cancer care before ISIL

According to the interviews conducted for this study, the oncology apparatus of Kirkuk was on a steady rise before ISIL. In 2007, a small public cancer unit with three oncologists and 12 beds was established within Azadi General Hospital in Kirkuk city center. The unit provided a limited range of diagnostics and basic chemotherapy (e.g., Adriamycin, Docetaxel, Paclitaxel, Vincristine). The patient load averaged 20-25 per day. In 2011 the oncology department expanded to occupy an entire floor of Azadi Hospital, and two oncologists were added to the team, totaling five. With the facility’s expansion to 50-60 beds, chemotherapy agents increased (e.g., Herceptin, Avastin, Rituximab). As a result, the oncology unit developed a strong enough reputation to attract some referrals from Baghdad, Nineveh, Diyala, and Tikrit in addition to the local patients. By 2013, the oncology unit was receiving 30-45 patients daily. However, there were still serious limitations due to the absence of radiotherapy and scarce access to CT scans. Still, cancer services were on a positive trajectory that outpaced other provinces such as Anbar. This development was crucial for Kirkuk citizens due to the distances required to reach other centers. Before the introduction of oncology in 2007, doctors in Kirkuk referred patients diagnosed with cancer to Baghdad (275 kilometers to the south) or Mosul (175 kilometers to the northwest) for chemotherapy and radiotherapy.

#### 3.3.3 Cancer care during ISIL

ISIL never overtook the urban center of Kirkuk, where the public oncology unit is located. In 2014, ISIL established a presence across the southern areas of Kirkuk province (e.g., Hawija district) but never succeeded in overtaking Kirkuk city. The relative stability in Kirkuk city led to an influx of displaced persons – including cancer patients. During 2014 and 2015, the daily patient load increased dramatically to 120/day. Most displaced patients arriving from the peripheries of the province and neighboring governorates lacked pathology reports and imaging. As there were only 2 CT scans in the city and one MRI machine, oncologists increasingly relied upon a mixture of X-rays, ultrasounds, and blood tests.

Fortunately, the MoH in Baghdad recognized the massive burden of displaced patients and decided to transfer large portions of the pharmaceutical stocks designated for Nineveh, Salah al-Din and Diyala provinces to Kirkuk. Because of this policy, important chemotherapy agents (e.g., Herceptin, Avastin, Cisplatin, Carboplatin) and hormonal therapy remained available throughout the ISIL conflict. Oncologists look back upon the ISIL period as, ironically, the time when crucial medications were most reliably present.

But other aspects of cancer care remained stagnant or worsened during the ISIL conflict. The lack of radiotherapy in Kirkuk created serious challenges during the ISIL period. Kirkuk’s oncologists had for many years referred local patients needing radiotherapy to Baghdad, Sulaymaniyah, or Erbil. But after the rise of ISIL, patients were repeatedly turned back at the checkpoints entering neighboring provinces. These rejections were especially common for displaced cancer patients whose places of origin were the districts and provinces then occupied by ISIL. As a hub for many displaced cancer patients, Kirkuk oncologists had to manage the complex and painful therapeutic trajectories of patients who were being denied access to referrals and had to face either curative or palliative care without radiotherapy.

Though the MoH’s conflict management of pharmaceuticals was viewed favorably in Kirkuk, other policy decisions during the conflict were more controversial. As Azadi General Hospital became a cross-provincial hub for oncology during the ISIL war, specialists in other disciplines complained that Kirkuk lacked adequate space to manage such an influx of cancer patients. This prompted the MoH in August 2016 to establish a separate unit for oncology in a building 500 meters across the road from the main hospital. While this decision reduced overcrowding at Azadi, some oncologists feel it created onerous physical and administrative barriers to accessing multi-disciplinary consultations and diagnostic services.

Kirkuk’s centrality as a conflict oncology hub would become less prominent in the war’s later stages. In December 2016, an ISIL attack in central Kirkuk prompted local security forces to expel scores of Sunni Arab displaced persons from the provincial capital on the unfounded pretext that their ongoing presence in the city constituted a security threat and that their cities of origin were now free from ISIL. The forced expulsions immediately impacted cancer patient levels. In the coming weeks and months, the patient load at the newly established oncology center went from 120/day to 40/day. Meanwhile the expelled cancer patients struggled to piece together care in other Iraqi cities or abroad – often at great personal expense.

#### 3.3.4 Cancer care after ISIL

The liberation of southern Kirkuk province in October 2017 generated a final surge of complex and advanced cases in the oncology center. From that point onward, cancer patient levels have gradually returned to pre-ISIL levels (30-40 patients/day). It is now rare to see patients from the neighboring provinces of Anbar, Nineveh, and Diyala in Kirkuk. Kirkuk’s loss of its hub-like status is partly a product of the waning of conflict and the return of populations to places of origin. It is also a product of the stagnation in oncology services. Unlike Anbar, which has witnessed a dramatic expansion of oncology services and human resources, Kirkuk levels have remained constant. There were 5 oncologists in Kirkuk before, during, and after the conflict. Only in the past 12 months (2022) have an additional 3 oncologists been added to the team. Pharmaceutical stocks – which again were strong during the conflict years – have regressed. Herceptin, commonly available in 2013 (the year before ISIL) and throughout the conflict (2014-2017), is only rarely in stock. Basic diagnostics such as CT scans remain exceedingly scarce and must usually be acquired privately. There are no plans to establish radiotherapy services in Kirkuk, forcing patients to travel across provinces or borders for treatment relying on personal finances. The discussion section will address the political dynamics undergirding the stagnation of oncology services in Kirkuk.

### 3.4 Salah al-Din Province

Limited oncology services were initiated in the provincial capital of Tikrit in 2008 under the direction of one oncologist. This oncology unit in Tikrit had minimal capacity and primarily received patients from the capital city and the immediate surrounding districts (e.g., Tikrit, Al-alam, Beiji). Patients from southern regions of the province (e.g., Balad and Dijeil districts) continued to seek cancer care in Baghdad, while patients in the northern regions (e.g., Shirqat, Tuz Khormato) sought care in Mosul and Erbil. When ISIL took over Tikrit in 2014, cancer services in Tikrit were suspended but were soon reopened in Samarra, a southwestern district of the province that had not fallen to ISIL. Oncology services were delivered in Samarra city at minimum capacity throughout the ISIL occupation of Tikrit. Oncology services were reconstituted in the capital city by the beginning of 2016, now with two oncologists. Cancer services have remained limited between the time of liberation and the present. However, to the surprise of the local medical establishment, the provincial government recently announced plans for a new oncology center that would include a linac machine. Why the provincial government (not the MoH) has decided to take the lead on this initiative remains unknown. If completed, the center would be the only oncology facility in the post-ISIL territories with a working linac machine.

### 3.5 Diyala Province

Diyala did not have oncology services of any kind before ISIL nor during the conflict. In the aftermath of liberation, a small department was created in 2018 under the supervision of one oncologist. At the time, the department lacked the approval of the MoH for the provision of chemotherapy from the public stocks. Thus, it relied exclusively on the transfer of drugs from cancer centers in other provinces. Through the advocacy efforts of local staff, chemotherapy approval was obtained in 2019, and a stand-alone center is currently under construction. At the moment, the oncology unit is staffed by two oncologists. It is primarily dedicated to the administration of chemotherapy and hormonal therapy, and it lacks diagnostic scanning, genetic tests, immunohistochemistry, immunological therapy, biological therapy, and radiotherapy. The new center is anticipated to include a broader range of diagnostic and therapeutic capacities.

## 4 Discussion

The aim of this article has been to highlight the impact of war on oncology infrastructure in five Iraqi provinces, specifically those that were impacted directly by the recent ISIL conflict. Examining each of the five provinces across the three periods (Before ISIL, During ISIL, After ISIL) has revealed important commonalities and differences in the experience of conflict at the level of cancer care services. These commonalities and differences are explored in greater detail in the following sections.

### 4.1 Common Challenges Across the Five Provinces

#### 4.1.1 Gaps in cancer data/registry

An extensive analysis of the epidemiological data from each of the five provinces is not included in this study due to a lack of reliability in the data. The cancer registry in Iraq, including the overall data management system, has suffered under protracted conflict (20). Particularly in the five provinces affected by the ISIL conflict, war-induced displacements of cancer patients as well as the phenomenon of cross-border travel for care have exacerbated overall gaps in Iraq’s cancer registry. **Table 2** summarizes the MoH cancer data in the five provinces compared to country-wide averages for the year 2020 (21). Both the crude incidence rate (CIR) and distribution of the top ten cancers are of questionable reliability due to conflict-related displacement, migration, and cross-border care-seeking patterns. It is worth noting that the cancer of unknown primary (CUP) is among the top ten cancers in Nineveh in 2020, unlike the status ten years ago, in 2009 (22) nor even twenty years ago, in 1999 (23). Though CUP globally can range from 2.3% to 5% (24), the existence of a 3.94% CUP rate in Nineveh – compared to CUP in Erbil, Basra, Karbala, or in Iraq as a whole, where the rates were 2.53%, 2.44%, 1.01%, and 2.63%, in sequence – is a clear indication of the conflict-related difficulties and degradation of cancer diagnostic capacity that led to the higher rate of CUP in Ninevah in the recent years.

**Table 2:** Cancer data from the five provinces compared to the whole country, extracted from the 2020 cancer registry (21).

	Nineveh	Anbar	Kirkuk	Salah al-Din	Diyala	IRAQ
**Population**	3926931	186416	168346	1678554	1722983	40150200
**New cases**	2511	1212	956	1083	1281	31692
**CIR**	63.94/100000	65/100000	56.8/100000	64.52/100000	74.35/1000000	78.93/100000
**1^st^ Top ten**	Breast	Breast	Breast	Breast	Breast	Breast
**2^nd^ Top ten**	Lung	Thyroid	CRC	Thyroid	CNS	Lung
**3^rd^ Top ten**	CRC	CRC	Lung	Lung	Lung	CRC
**4^th^ Top ten**	CNS	Lung	CNC	CRC	CRC	CNS
**5^th^ Top ten**	Leukemia	NHL	NHL	Leukemia	Leukemia	Leukemia
**6^th^ Top ten**	Stomach	HL	Prostate	CNC	Stomach	Urinary bladder
**7^th^ Top ten**	Thyroid	Leukemia	Leukemia	Urinary bladder	Prostate	Thyroid
**8^th^ Top ten**	NHL	CNC	Kidney	Stomach	Thyroid	NHL
**9^th^ Top ten**	CUP	Stomach	Ovary	Skin	Skin	Prostate
**10^th^ Top ten**	Skin	Urinary bladder	Uterus	Uterus	Urinary bladder	Stomach
**Top ten no. (%)**	1687 (67.18%)	877 (72.36%)	671 (70.19%)	747 (69.98%)	869 (67.84%)	20874 (65.87%)

CIR, Crude incidence rate; CNS, central nervous system; CRC, colorectal cancers; CUP, cancer of unknown primary; HL, Hodgkin’s Lymphoma; NHL, Non-Hodgkin’s Lymphoma.

#### 4.1.2 Human resources

Across the five provinces examined in this study, maintaining the necessary human resources in medical and radiation oncology has been a persistent challenge (14). At the country-wide level, the origin of this problem dates to the 2003 US-led invasion and occupation, when many highly experienced doctors fled the country due to threats, intimidation, and violence (15). The 2014-2017 ISIL conflict displaced many oncologists and placed heavy burdens on those who remained in place. Over the past five years, progress has been made towards developing cohorts of local oncology specialists, but persistent gaps remain. The slow pace of reconstruction and lack of resources directed towards public oncology centers has increasingly incentivized both cancer specialists and nurses to allocate time to private clinics. Though private clinics may expand access for some patients in the short-term, the solidification of this trend may also reduce the quality and availability of public oncology to a vulnerable war-affected population.

#### 4.1.3 Lack of radiotherapy

As noted in the results, the persistent lack of radiotherapy has become a glaring issue across the five provinces impacted by the ISIL conflict. While other provinces in the South and Kurdistan region have witnessed an increase in radiotherapy capacity in recent years (25), the five provinces included in this study have yet to see any progress in this domain. By the end of 2022, there were a total of 34 functional linacs for radiotherapy (25 linacs are public and 9 are private): 8 in the federal capital, Baghdad, 5 in Kurdistan’s three provinces, and 21 in the nine provinces south of Baghdad. None were available in the five provinces highlighted in this study (25).

#### 4.1.4 Pharmaceutical supply

In theory, the Iraqi MoH provides cancer drugs and chemotherapy free of charge to all Iraqi citizens. But in practice, pharmaceutical supply has proven to be unreliable, particularly during heightened episodes of conflict. During the ISIL period (2014-2017), shortages in pharmaceuticals forced patients to rely either upon private pharmacies and/or the black market to purchase chemotherapy and other medications. The slow pace of oncology reconstruction in some provinces has exacerbated gaps in the public system and buoyed the private pharmaceutical market. Recent policy research has shown that increasingly unchecked privatization and endemic corruption has led to a proliferation of fake and expired medications (26), deepening citizens’ overall distrust of medicine.

#### 4.1.5 Diagnostic capacity

As has been identified in other areas of the Middle East facing protracted conflict (27), diagnostic capacity in Iraq remains weak and is a major reason why so many cancer patients travel abroad for care. With limited cross sectional anatomical studies (CT and MRI scanners) and absent nuclear medicine studies, particularly during acute phases of conflict, oncologists in the five provinces examined have too often been forced to piece together a diagnosis by relying upon a patchwork of simple X-rays, ultrasounds, and blood tests, compared to other provinces that did not pass through such ISIL conflict and had showed remarkable progress in cancer diagnostics (14, 27, 28).

#### 4.1.6 Cross-border & high-cost cancer journeys

Cancer patients have managed the lack of radiotherapy and other gaps in cancer services through cross-provincial treatment journeys in addition to international travel across borders. Between 2014 and 2017, cross-provincial referrals posed a significant risk for cancer patients from the ISIL-occupied provinces, who were repeatedly denied passage or interrogated by state security forces at checkpoints (11). Over the past five years, arbitrary denials at checkpoints have become less common, but nonetheless, the reliance upon travel for radiotherapy and other cancer services places profound practical and financial burdens upon Iraqi families. Cancer patients from a wide range of regions and socio-economic circumstances continue travelling to Iraq’s oncology hubs (e.g., Baghdad, Sulaymaniyah and Karbala) and to international private centers (e.g., Beirut, Istanbul, Amman) for cancer diagnostics and treatments, often selling homes and properties to fund lengthy periods of treatment away from home. For a cancer patient population that is already struggling to rebuild their homes and lives after war, the extra cost of cross-provincial and cross-border travel represents a catastrophic financial burden (12).

#### 4.1.7 Uneven distribution of care (urban/rural)

As a rule, cancer services are concentrated in the provincial capitals and remain totally absent in towns and mid-sized cities. For cancer patients residing in the rural areas of geographically vast provinces such as Anbar and Nineveh, the journey to the provincial capital for oncology is itself a major hardship and an expression of a glaring urban/rural divide. The urban/rural divide in medical infrastructure prolongs displacement, with displaced families often remaining in urban areas to retain access to oncology and other specialized medical services.

### 4.2 Variations in the Impact of War on Oncology Across Five Provinces

Though the shared challenges facing cancer services discussed above are vast, researchers must caution against an overly general narrative. Indeed, the impacts of war on oncology have locally distinct dimensions. These distinctions are highlighted in the following political economy analysis.

#### 4.2.1 Before ISIL

Before the rise of ISIL, the level of advancement in oncology was widely divergent across the five provinces studied. By 2013 (the year before the takeover of ISIL), Nineveh was home to well-established advanced oncology services; in Anbar, Salah al-Din, and Kirkuk, new oncology structures were emerging to varying degrees; in Diyala, no progress had been made. The uneven distribution of oncology resources across the five provinces reflected Iraq’s gradual transition from a highly centralized care delivery model to a partially federalized one. As early as the 1980s, Mosul (alongside Baghdad and Basra) witnessed the emergence of comprehensive high-level services because the MoH designed the national healthcare apparatus to channel tertiary referrals to the country’s three largest cities. This coordinated system of referrals continued but became less sustainable after the US-led invasion of 2003. Not only did Iraq’s hospitals in major cities suffer under the weight of violence and the proliferation of corruption, but the enshrinement of federalism in the 2005 constitution also encouraged the development of more localized medical infrastructures. Therefore, an increasing number of provinces (e.g., Kirkuk, Anbar, Salah al-Din, Erbil, Sulaymaniyah, etc.) witnessed the gradual emergence of oncology from 2007 onward. The progress towards localized care would not be seamless, however. The introduction of the federal model coincided with the emergence of political parties that exerted control over specific provinces (29, 30), which obscured the coherence of health governance at the local level.

#### 4.2.2 During the ISIL period

Oncology witnessed different impacts of the ISIL conflict depending on the locality. In Mosul, ISIL allowed the city’s well-established oncology services to continue basic operations partly because the group saw the city as its capital and wanted to maintain hospitals to treat the core of its fighting force. But cutting off supply lines to the city led to the gradual deterioration of public oncology and the emergence of a chaotic *ad hoc* system reliant upon private purchases and smuggling of drugs – at great cost for local cancer patients. In Anbar, nascent oncology services were curtailed entirely once ISIL took over the portion of the city where the oncology department was located due to the forced displacement of medical personnel. The relative proximity of Baghdad’s sizeable medical infrastructure meant that many of Anbar’s cancer patients could resume care in the capital city. Still, those who remained in the province suffered from a total lack of access to care. The irony of the evolution of oncology in Kirkuk is that the ISIL period arguably represented the pinnacle of its advancement and the height of its challenges. Oncology services were under extreme duress (due to the influx of displaced patients from surrounding provinces), and yet oncology services arguably reached their highest levels of progression (due to the shift of budgetary support towards pharmaceuticals from other provinces).

#### 4.2.3 After the ISIL period

Understanding the highly variable pace of oncology reconstruction requires close attention to political economic dynamics. Recent research in policy and political science has shown that the military offensive against ISIL reshaped the political composition of different provinces in significant ways, directly impacting the pace of reconstruction (30). In Nineveh and Kirkuk, the military campaign against ISIL led to a fragmentation of the local political order and the proliferation of factions competing for influence (30, 31). The resulting lack of coherence at the level of governance has slowed the pace of reconstruction in both provinces (30), with important implications for the rebuilding of oncology infrastructure and the delivery of cancer services. In Nineveh, the rebuilding of oncology facilities has witnessed repeated delays and oncologists continue to work in makeshift structures. In Kirkuk, the level of cancer care delivery has stagnated amid a lack of consensus among competing local political factions as well as reductions of government-provided pharmaceuticals.

In contrast, the reconstruction of oncology has proceeded relatively quickly in Anbar, in part due to the relative coherence of the political environment. Though political dynamics in Anbar are by no means simple (32), it was the sole province where a single political coalition emerged in the wake of the ISIL defeat that gained control over the local government as well as influential positions at the federal government level. Consequently, the various governmental authorities overseeing healthcare (e.g., the MoH, the Anbar Directorate of Health, and the Anbar provincial government) have been able to work relatively effectively together to restore and expand oncology. Encouraged by political stability, economic activity in Anbar spiked in the post-liberation era due to heavy foreign investment (33, 34). Though these foreign funds were not directly allocated towards public oncology institutions, they contributed to the overall rebuilding of the province’s housing units and economy, which enabled the speedy return of both the general population and oncology specialists.

This is not to say that post-conflict political economy is the only lens through which one can explain the variable pace of reconstruction. Differences in the level of war-related infrastructural damage have also played a role. While the aerial bombing was a feature of the liberation campaign in all major anti-ISIL battles, including Ramadi, there was arguably no destruction as total as what was witnessed in western Mosul, the site of Nineveh’s oncology infrastructure. The strategy of the Iraqi Security Forces and the US-led Coalition was to encircle and isolate ISIL into the western segment. The targeting practices that followed showed little signs of meaningful discrimination between military structures and civilian spaces, resulting in scores of civilian deaths (35). With most of the roads on the western side destroyed or badly damaged, supplying reconstruction projects was a challenge in and of itself. Healthcare reconstruction has languished, generating enormous challenges in access to care (36).

### 4.3 Implications for cancer research in regions of war

#### 4.3.1 Strengthening research on local cancer care delivery

A growing body of research is exploring the impact of war on oncology in the Middle East and across regions under conflict (37, 38). The bulk of this literature has viewed the understanding of cancer under conflict through a humanitarian lens (39), with particular attention paid to cancer care for refugees and displaced persons (40–42). These studies have highlighted the enormously complicated and fragmentary nature of treatment pathways for cancer care under conflict, such as “being unfamiliar with the health system, delays in health-seeking behavior because of competing priorities, financial limitations, or fear of persecution because of political or security issues related to their situation” (41). The humanitarian research lens is important and deserves resources and attention; however, in Iraq and many conflict-affected contexts, the medical infrastructures that absorb the bulk of cancer care needs during and especially after the formal cessation of conflict are local public oncology hospitals, which are too often ignored by humanitarian medicine and research.

By analyzing the impact of war on public cancer hospitals in five Iraqi provinces, this article connects the dots between the ‘conflict’ period (in which the humanitarian sector plays an important role) and the ‘post-conflict’ period (in which local hospitals are the centerpiece of care). Building upon prior work that has stressed the need for cancer research capacity development in the conflict-affected regions of the MENA (43), this study emphasizes the need for more research that focuses on locally specific care delivery dynamics in conflict-affected regions. In the Iraqi context, partnerships between the country’s public oncology centers and international research bodies can facilitate this effort.

#### 4.3.2 Strengthening epidemiological and environmental research

Though the focus of this article has been on the impact of war on cancer care and oncology infrastructure, it should also be noted that in each of these five provinces there are ongoing concerns and debates over the impact of war on the environment and the implications for cancer incidence. The US usage of depleted uranium munitions both in the 1990s and during the 2003-2011 period led to several important but methodologically limited studies examining potential linkages to the prevalence of certain cancers and other health conditions (5–7); however, such research has lacked the necessary resources and sustained support to establish clarity on this issue both at the national level and in distinct localities. Enduring questions over the impact of environmental exposure on cancer incidence will linger so long as local cancer research institutions lack the necessary funding to conduct interdisciplinary epidemiological and environmental studies, ideally in collaboration with partners across the Middle East region facing similar legacies of war.

### 4.4 Implications for Cancer Policy

#### 4.4.1 Navigating oncology reconstruction amid complex local realities

Regarding the challenge of oncology reconstruction in the wake of conflict, the article indicates that the best-laid national cancer care plans must face political and economic realities at the national and sub-national levels. War disrupts and fragments local political orders in ways that endure long after formal hostilities have ceased. In the Iraqi context, provinces with more political fragmentation in the aftermath of the ISIL conflict have also witnessed a slower road to recovery in oncology and the broader medical infrastructure. In such fraught environments, the reconstruction of oncology requires building support and agreement among competing political groups and other local stakeholders. To that end, developing a granular understanding of the post-conflict political economy must be an integral part of the cancer policy formulation and implementation process.

#### 4.4.2 Supporting public oncology institutions

Though addressing the gaps in cancer care described in this article will require the efforts of a broad set of stakeholders and diverse policy approaches, the growing calls for the greater privatization of medicine in Iraq should be received with caution. War has generated gaps in public medicine that are increasingly filled by a fragmented array of private services in Iraq and neighboring countries. As a direct consequence of this trend, catastrophic medical expenditure has become a major problem for families struggling to piece back their lives in the wake of conflict. Though the further expansion of private care is probably inevitable and may improve access to oncology for some patients, the hope of most Iraqi oncologists is for the government and international organizations to support them in strengthening public oncology hospitals across the country’s 19 provinces, equipping these institutions with the capacity to provide comprehensive care during the current period of post-conflict recovery and relative stability, and in the face of whatever future wars may come.

## 5 Conclusion

Previous studies have called attention to the devastating impact of war on Iraq’s once robust public oncology infrastructure. While this research has shown important trends in the war-induced deterioration of specific cancer services, it has yet to explain the uneven impacts of conflict across different regions and localities. The aim of this study has been to contextualize and understand local variations in the impact of war on oncology and the pace of reconstruction. The operational capacity of public cancer services in the five provinces directly impacted by the ISIL conflict (Nineveh, Anbar, Kirkuk, Salah al-Din and Diyala) was analyzed across three periods — before, during, and after the ISIL conflict. The major claim of the study is that conflict generates both immediate and long-term political and economic dynamics that contribute to locally specific divergences in the delivery of care and pace of reconstruction. Departing from the humanitarian focus of prior research on cancer under conflict, the article calls for increased scholarly attention to the variable capacities of public hospitals impacted by war — as they are the oncology infrastructures serving conflict-affected patients both during and long after hostilities formally cease.

## Data availability statement

The original contributions presented in the study are included in the article/supplementary material. Further inquiries can be directed to the corresponding author.

## Author contributions

The first author and the corresponding author are the owners of this project. Both the first author and corresponding author drafted the manuscript and arranged the interviews. All authors contributed to the article and approved the submitted version.

## References

[1] Mula-Hussain L Alabedi H Al-Alloosh F Alharganee A. Cancer in war-torn countries: Iraq as an example. In: Laher I, editor. Handbook of healthcare in the Arab world. Cham: Springer International Publishing (2019). p. 1–14. doi: 10.1007/978-3-319-74365-3_152-1

[2] Alwan N Kerr D. Cancer control in war-torn Iraq. Lancet Oncol (2018) 19(3):291–2. 10.1016/S1470-2045(18)30135-9 29508747

[3] Al-Ghazi M. Cancer care in a war zone: Radiation oncology in Iraq. Int J Radiat Oncol Biol Phys (2016) 96(2, Supplement):E413.

[4] Mula-Hussain L Al-Ghazi M. Cancer care in times of war: radiation oncology in Iraq. Int J Radiat Oncol Biol Phys (2020) 108(3):523–9. 10.1016/j.ijrobp.2020.05.060 32976784

[5] Al-Hadithi TS Al-Diwan JK Saleh AM Shabila NP. Birth defects in Iraq and the plausibility of environmental exposure: A review. Confl Health (2012) 6(1):3. 22839108 10.1186/1752-1505-6-3 PMC3492088

[6] Fathi RA Matti LY Al-Salih HS Godbold D. Environmental pollution by depleted uranium in Iraq with special reference to mosul and possible effects on cancer and birth defect rates. Med Confl Surviv (2013) 29(1):7–25. 23729095 10.1080/13623699.2013.765173

[7] Aitken M. Gulf war leaves legacy of cancer. BMJ. (1999) 319(7207):401–1. 10.1136/bmj.319.7207.401a PMC1127036 10445914

[8] Al-Hadad SA Al-Jadiry MF Al-Darraji AF Al-Saeed RM Al-Badr SF Ghali HH. Reality of pediatric cancer in Iraq. J Pediatr Hematol Oncol (2011) 33 Suppl 2:S154–6. 10.1097/MPH.0b013e318230e218 21952575

[9] Fadhil SA Ghali HH. The current situation of palliative care services in Iraq. In: Palliative care for chronic cancer patients in the community. Cham: Springer International Publishing (2021). p. 341–9.

[10] Dewachi O Skelton M Nguyen VK Fouad FM Sitta GA Maasri Z. Changing therapeutic geographies of the Iraqi and Syrian wars. Lancet (2014) 383(9915):449–57. 10.1016/S0140-6736(13)62299-0 24452046

[11] Skelton M Mula-Hussain LYI Namiq KF. Oncology in iraq’s Kurdish region: Navigating cancer, war, and displacement. J Glob Oncol (2017) 4):1–4. doi: 10.1200/JGO.2016.008193 PMC6180800 30241196

[12] Skelton M Alameddine R Saifi O Hammoud M Zorkot M Daher M. High-cost cancer treatment across borders in conflict zones: Experience of Iraqi patients in Lebanon. JCO Glob Oncol (2020) 6:59–66. 32031440 10.1200/JGO.19.00281 PMC6998032

[13] Al-Ibraheem A Abdlkadir A. Theranostics in the Arab world; achievements & challenges. Jordan Med J (2022) 56(2):188–205.

[14] Al Alwan NAS. General oncology care in Iraq. In: Al-Shamsi H Abu-Gheida I Iqbal F Al-Awadhi A, editors. Cancer in the Arab world, 1st ed. Singapore: Springer Singapore (2022). p. 63–82. Available at: file:///Users/Layth/Downloads/978-981-16-7945-2_5.pdf.

[15] Burnham GM Lafta R Doocy S. Doctors leaving 12 tertiary hospitals in Iraq, 2004–2007. Soc Sci Med (2009) 69(2):172–7. 10.1016/j.socscimed.2009.05.021 19501443

[16] Dewachi O. Ungovernable life: mandatory medicine and statecraft in Iraq. Stanford: Stanford University Press (2017).

[17] Lafta RK Al-Nuaimi MA. War or health: a four-decade armed conflict in Iraq. Med Confl Surviv (2019) 35(3):209–26. 10.1080/13623699.2019.1670431 31597450

[18] Caglevic C Rolfo C Gil-Bazo I Cardona A Sapunar J Hirsch FR. The armed conflict and the impact on patients with cancer in Ukraine: Urgent considerations. JCO Glob Oncol (2022) 8:e2200123. 35994695 10.1200/GO.22.00123 PMC9470147

[19] Saleh S Fouad FM. Political economy of health in fragile and conflict-affected regions in the middle East and north Africa region. J Glob Health (2022) 12:01003. 35959965 10.7189/jogh.12.01003 PMC9373566

[20] Mula-Hussain L. Cancer care in Iraq: a descriptive study. 1st ed. Saarbrucken-Germany: LAP LAMBERT Academic Publishing (2012).

[21] Iraqi Cancer Registry. 2020 Annual Iraqi cancer report. Baghdad: Ministry of Health (2022).

[22] Iraqi Cancer Board. 2009 Annual Iraqi cancer report. Baghdad: Ministry of Health (2012).

[23] Iraqi Cancer Board. 1999 Annual Iraqi cancer report Baghdad: Ministry of Health (2002).

[24] Qaseem A Usman N Jayaraj JS Janapala RN Kashif T. Cancer of unknown primary: a review on clinical guidelines in the development and targeted management of patients with the unknown primary site. Cureus (2019) 11(9):e5552. doi: 10.7759/cureus.5552 31695975 PMC6820325

[25] Mula-Hussain L. Update about radiation status and demand in Iraq. In: 1st MESTRO (Middle-East society for therapeutic radiation oncology) conference. Riyadh: Alfaisal University (2022).

[26] Mansour R. Moving medicine in Iraq: networks fuelling everyday conflict. UK: The Royal Institute of International Affairs (Chatham House) (2022). Available at: https://www.chathamhouse.org/2022/11/moving-medicine-iraq-networks-fuelling-everyday-conflict.

[27] Al-Ibraheem A Abdlkadir AS Mohamedkhair A Mikhail-Lette M Al-Qudah M Paez D. Cancer diagnosis in areas of conflict. Front Oncol (2022) 12. 10.3389/fonc.2022.1087476 PMC9815758 36620568

[28] Paez D Becic T Bhonsle U Jalilian AR Nuñez-Miller R Osso JA. Current status of nuclear medicine practice in the middle East. Semin Nucl Med (2016) 46(4):265–72. 10.1053/j.semnuclmed.2016.01.005 27237437

[29] Saleem ZA. The king of salah al-din: The power of iraq’s Sunni elites. London: Conflict Research Programme, The London School of Economics & Political Science (2021). Available at: http://eprints.lse.ac.uk/108541/1/Ali_Saleem_the_king_of_salah_al_din_published.pdf.

[30] Skelton M Saleem ZA. Iraq’s political marketplace at the subnational level: The struggle for power in three provinces. London: Conflict Research Programme, The London School of Economics & Political Science (2020). Available at: http://eprints.lse.ac.uk/105184/3/Iraq_s_Political_Marketplace.pdf.

[31] Hasan Hama H Hassan Abdulla F. Kurdistan’s referendum: The withdrawal of the Kurdish forces in kirkuk. Asian Aff (Lond) (2019) 50(3):364–83.

[32] Hodge J Gopal A. The rise of the new Sunni elite in Iraq: The case of fallujah. UK: Conflict Research Programme, The London School of Economics & Political Science (2020). Available at: https://blogs.lse.ac.uk/crp/2020/11/16/the-rise-of-the-new-sunni-elite-in-iraq-the-case-of-fallujah/.

[33] Foltyn S. Anbar’s post-ISIL reconstruction spawns autonomy debate. Qatar: Al Jazeera (2021). Available at: https://www.aljazeera.com/economy/2021/1/27/iraq-anbars-post-isil-reconstruction-spawns-autonomy-debate.

[34] Cornish C. Fallujah land’: Iraq’s anbar province rebuilds after ISIS. UK: Financial Times (2021). Available at: https://www.ft.com/content/c1524f45-96ee-4323-bacf-9dd80d3287bb.

[35] Lafta R Al-Nuaimi MA Burnham G. Injury and death during the ISIS occupation of mosul and its liberation: Results from a 40-cluster household survey. PloS Med (2018) 15(5):e1002567. 29763433 10.1371/journal.pmed.1002567 PMC5953440

[36] Lafta R Al-Nuaimib MA Sultan LR Rihawa H Burnham G. Health care and care-seeking in mosul 1 year after defeat of ISIS. Disaster Med Public Health Prep (2022) 16(4):1524–31. 10.1017/dmp.2021.112 34284845

[37] Yusuf MA Hussain F Sultan F Badar F Sullivan R. Cancer care in times of conflict: Cross border care in Pakistan of patients from Afghanistan. Ecancermedicalscience (2020) 14. 10.3332/ecancer.2020.1018 PMC7105336 32256701

[38] Jawad M Millett C Sullivan R Alturki F Roberts B Vamos EP. The impact of armed conflict on cancer among civilian populations in low- and middle-income countries: A systematic review. Ecancermedicalscience (2020) 14. 10.3332/ecancer.2020.1039 PMC7289611 32565892

[39] Aebischer Perone S Martinez E du Mortier S Rossi R Pahud M Urbaniak V. Non-communicable diseases in humanitarian settings: Ten essential questions. Confl Health (2017) 11(1):17. 28932259 10.1186/s13031-017-0119-8 PMC5602789

[40] Abdul-Khalek RA Guo P Sharp F Gheorghe A Shamieh O Kutluk T. The economic burden of cancer care for Syrian refugees: A population-based modelling study. Lancet Oncol (2020) 21(5):637–44. 10.1016/S1470-2045(20)30067-X 32359488

[41] Spiegel P Khalifa A Mateen FJ. Cancer in refugees in Jordan and Syria between 2009 and 2012: Challenges and the way forward in humanitarian emergencies. Lancet Oncol (2014) 15(7):e290–7. 10.1016/S1470-2045(14)70067-1 24872112

[42] El Saghir NS Soto Pérez de Celis E Fares JE Sullivan R. Cancer care for refugees and displaced populations: Middle East conflicts and global natural disasters. Alexandria, VA: American Society of Clinical Oncology Educational Book (2018) p. 433–40. 10.1200/EDBK_201365 30231320

[43] Abdul-Sater Z Kobeissi E Menassa M Telvizian T Mukherji D. Research capacity and training needs for cancer in conflict-affected MENA countries. Ann Glob Health (2020) 86(1):142. 33200073 10.5334/aogh.2809 PMC7646279

